# Online Symptom Checkers: Recommendations for a Vignette-Based Clinical Evaluation Standard

**DOI:** 10.2196/37408

**Published:** 2022-10-26

**Authors:** Annabelle Painter, Benedict Hayhoe, Eva Riboli-Sasco, Austen El-Osta

**Affiliations:** 1 Self-Care Academic Research Unit Department of Primary Care and Public Health Imperial College London London United Kingdom

**Keywords:** online symptom checkers, clinical evaluation, validation, assessment, standards, third-party assessment, quality assurance

## Abstract

The use of patient-facing online symptom checkers (OSCs) has expanded in recent years, but their accuracy, safety, and impact on patient behaviors and health care systems remain unclear. The lack of a standardized process of clinical evaluation has resulted in significant variation in approaches to OSC validation and evaluation. The aim of this paper is to characterize a set of congruent requirements for a standardized vignette-based clinical evaluation process of OSCs. Discrepancies in the findings of comparative studies to date suggest that different steps in OSC evaluation methodology can significantly influence outcomes. A standardized process with a clear specification for vignette-based clinical evaluation is urgently needed to guide developers and facilitate the objective comparison of OSCs. We propose 15 recommendation requirements for an OSC evaluation standard. A third-party evaluation process and protocols for prospective real-world evidence studies should also be prioritized to quality assure OSC assessment.

## Introduction

The last decade has seen a proliferation of online symptom checkers (OSCs). The pervasiveness of smartphones, tablets, and personal computers has increased the availability of these free and accessible decision support tools that offer on-demand symptom assessment at scale [1]. Although many OSCs products are developed by commercial companies as direct-to-consumer products, several products have been deployed within national health care systems including ‘National Health Service (NHS) 111’ online and Babylon ‘Ask A&E’ in the United Kingdom and ‘healthdirect’ in Australia. These patient-facing general purpose symptom checkers are intended for members of the public to use at home as a decision aid to help inform them about the potential cause of their symptoms and where to seek care.

Despite widespread use of OSCs, there are various concerns about their clinical safety and accuracy [1-7]. A key factor contributing to this uncertainty stems from a lack of consensus regarding an objective methodology or an agreed standard for OSC evaluation.

Although OSCs must comply with Medical Device Regulations [8] and are encouraged to align with evidence standards [9], governance structures for digital health technologies in the United Kingdom and European Union do not stipulate any specific clinical evaluation method or protocol for OSCs [8,9]. In 2020, the Care Quality Commission conducted the first regulatory sandbox focused on digital triage tools, highlighting the following:

[D]igital triage tools are not fully clinically validated or tested by product regulators and notified bodies. We have learned that there is great variation in their clinical performance. [10]

Most OSCs are registered as Class I medical devices in the European Union and the United Kingdom [3]. Class I status involves self-certification by developers and does not require assessment by a notified body [8]. In the United Kingdom, part II of the UK Medical Device Regulations 2002 requires that Class I products must provide evidence of clinical evaluation and that “the data needs [*sic*] to adequately demonstrate that the product fulfils its intended purpose” [8]. However, neither the Medical Device Regulations nor the Medicines and Healthcare products Regulatory Agency provide detailed guidance on how this should be carried out for OSCs specifically, and they do not stipulate a requirement for objective third-party assessment. This has meant that developers can create their own internal methods for clinically evaluating their products without the need for an objective or impartial assessment to be undertaken.

The National Institute of Health and Care excellence (NICE) in the United Kingdom has published and recently updated a set of evidence standards for digital health technologies [9]. This guidance categorizes digital health technologies into various tiers and suggests appropriate evidence requirements for each. However, this guidance does not include any specific approaches for OSC clinical evaluation methodology.

The lack of consensus on OSC clinical evaluation methodology may also account for conflicting results reported in comparative research studies of OSCs [1,2,4-6,11,12]. Despite appearing to share a similar evaluation approach (eg, using clinical case vignettes to compare OSCs to a ‘gold standard’ set by clinicians), there is notable variation in the methods used at various steps of the evaluation processes in these studies. These include differences in the number, type, and content of vignettes used; who and how many people input the vignettes into OSCs; how the gold standard diagnostic and triage solutions are chosen; how results are benchmarked against the gold standard; as well as the number and specification of performance metrics used. Inconsistencies of study findings may be further compounded by the low quality of most comparative studies published to date, which are largely observational studies, usually published as grey literature and often by OSC developers themselves, introducing a significant risk of bias [7,13]. As a result, the findings of most vignette-based OSC studies are difficult to reproduce independently, and this applies especially to those studies published by OSC developers.

The need for more robust clinical evaluation guidelines for OSCs has been highlighted in existing literature [7,14]. Suggestions include applying extant evaluation frameworks currently used in mobile health and health informatics to OSCs [14]. Future recommendations should ideally build on these suggestions to inform the development of a standard for vignette-based OSC clinical evaluation methodology. The aim of this paper is to characterize a set of congruent requirements for a standardized OSC vignette-based clinical evaluation process.

The recommendations in this paper were developed through evaluation of primary literature alongside informal discussions with OSC developers involved in clinical evaluation and researchers who have undertaken comparative OSC studies.

## Recommended Requirements for an OSC Clinical Evaluation Standard

Robust clinical evaluation guidelines are required to align the processes used by both developers and evaluators of patient-facing general purpose OSCs. The development of a congruent and evidence-based guideline is needed to help provide assurance that OSCs are fit for purpose, promote patient safety, and can help facilitate objective and reliable product comparison and benchmarking.

The variability in results from comparative studies highlights the vulnerability of current vignette-based OSC evaluation approaches. Therefore, any standard for vignette-based clinical evaluation of OSCs will require careful consideration to ensure an objective and robust process is specified, including guidance on how these processes should be implemented and reported in a way that is open and transparent.

Table 1 summarizes 15 key requirements across 7 categories within vignette-based clinical evaluation methodology that could benefit from standardization. These recommendations are intended to guide the creation of a shared standard to be followed by both developers and researchers of OSCs when undertaking a clinical evaluation process using vignettes. Although individual vignette data sets and methodological details may vary according to a given OSC use case, this variation would be limited by the parameters set out in this proposed standard. These recommendations do not represent a standardized third-party evaluation protocol but could be followed in the design of such a process. Third-party benchmarking is discussed further in the ‘Recommendations for future work’ section.

**Table 1:** Summary of recommended requirements for a vignette-based online symptom checker (OSC) clinical evaluation standard.

Category	Requirements
Vignettes	Illustrate the method for determining the minimum number of vignettes required for OSC evaluation. Specify the minimum information to be included in each vignette. Provide guidance on determining the conditions, symptoms, or spread of cases to be included and assurance that this appropriately represents the target user population. Specify vignette origin requirements (eg, simulated vs real-world cases).
Clinician assessment	Specify the appropriate clinicians (including role, speciality, and seniority) to be used in the assignment of ‘gold standard’ labels. Illustrate the method used for compilation of a gold standard (eg, averaged single blinded assessment or consensus discussion).
Triage	Specify standardized triage categories, including setting and time periods. Provide guidance on the use of triage gold standards as a range for both urgency and setting.
Differential diagnosis	Illustrate the method for comparing OSC differential diagnosis list to gold standard dispositions.
Accuracy	Illustrate the accuracy and safety score calculation method, accounting for outcomes that fall both below and above the gold standard. Specify the minimum accepted accuracy and safety scores.
Safety netting	Specify how safety netting contributes to product safety scores.
Inputters	Specify the minimum interrater reliability scores for inputters of vignettes. Illustrate the method for determining the number of tests and inputters required for each vignette. Specify the appropriate characteristics for vignette inputters (eg, medical education level and affiliation with developers).

## Vignettes

OSCs are most often validated using a set of clinical case ‘vignettes’ [4-6,11,12]. Each vignette represents a possible clinical scenario or ‘case’ and contains information such as key patient demographics, relevant medical history, and symptoms. This method has also been used to assess the reliability of clinician-facing diagnostic decision support tools [15,16] and diagnoses made by clinicians [17,18].

The number of vignettes that should be used during clinical evaluation of an OSC is not defined. There is a lack of guidance about the spread of diseases or presentations that should be included in any given vignette set. There is no guidance on how representative the vignettes should be of the target user population in terms of disease incidence or prevalence and demographics, such as gender, age, and ethnicity, risking an increase in existing inequalities through a lack of inclusion [19]. The minimum level or amount of information that each individual vignette should contain (ie, user demographics, comorbidities, and current medications) is also undefined.

The use of vignettes for clinical evaluation has limitations. Clinicians assessing vignettes are restricted to the information provided without the opportunity to ask additional questions, examine the patient, or assess nonverbal cues. Meanwhile, when an OSC is being tested using a case vignette, the inputter may be forced to make assumptions when answering questions about aspects that are not illustrated in the finite vignette script [12].

Most published OSC comparative studies use disease-based vignettes authored by clinicians [4,5,11,12,16]. These vignettes have additional constraints as clinical authors are likely to describe symptoms differently to patients and vice versa. These imagined vignettes are also subject to bias from authors’ clinical experience and education and may result in ‘textbook’ presentations of diseases rather than realistic cases. The potential for bias is further compounded by the fact that vignettes used in the clinical evaluation of an OSC by developers can be written by clinicians employed by developers themselves. Vignettes coproduced with direct patient input and based on real-world patient-reported symptoms that are not created by OSC developers may be preferable for use in OSC validation studies.

Given the limitations of a vignette-based approach, any OSC clinical evaluation standard involving vignettes should specify the following: (1) the number of case vignettes that must be used to test an OSC; (2) the minimum information to be contained in each vignette; (3) the conditions, symptoms, or spread of cases, that must be included in this data set, including representability to the target user population demographics; and (4) the provenance and creation process of the vignettes (eg, whether they are simulated or real-world cases).

## Clinical Assessment

### Gold Standard

A ‘gold standard’ label is a term used to refer to an ‘ideal’ set of outcomes to which an OSC is compared during a clinical evaluation process. In clinical practice, there is no ‘ideal’ way of triaging patients and no ‘perfect’ differential diagnosis. However, gold standard triage and diagnostic labels are required to obtain a quantitative assessment of OSC performance. The ‘gold standard’ vignette labels used in these assessments are generally assigned by practicing clinicians [4,5,12,20].

The type of clinical professional and the speciality and level of seniority of the clinicians used to generate these labels are all likely to have an impact on the gold standard that is generated. Published studies comparing OSCs to date have used different types and number of clinicians to develop their gold standard labels. Several studies used groups of general practitioners (GPs) [11,12], whereas others used a range of clinical professionals including GPs, paramedics, pharmacists, emergency medicine consultants, and triage nurses [1,4,20]. These differences may have contributed to the varying outcomes of these studies and highlights the need for a consensus.

Gold standard labels are vulnerable to significant interclinician variability even among clinicians from the same field of specialization [12,17,18]. Therefore, the method in which the labels from different clinicians are collated can impact the gold standard. There is a notable disparity in the collation methods used in published studies. Whereas some studies collate assessments by using the majority outcome or the most severe outcome [11,20], other approaches center around asking clinicians to discuss cases together to reach a consensus decision either in a single session or following a series of ‘roundtables’ [1,12].

Variability in the clinician type and number as well as the methods used to assign and compile gold standards is likely to have contributed to the inconsistency in the results of published OSC evaluation studies. A vignette-based OSC clinical evaluation standard should specify the appropriate cadre of clinicians (including role, speciality, and seniority) and the approach they use to assign gold standard labels and illustrate the method for compilation of a gold standard (eg, averaged single blinded assessment or consensus discussion).

## Triage

OSCs may provide users with a triage or priority recommendation advising at what setting and with what degree of urgency to seek help. Urgency refers to how soon a person should be assessed by a health care professional (eg, seek care immediately, within 48 hours, or within 3 weeks), whereas setting refers to the specific area of health care most appropriate for this assessment (eg, emergency department, GP, or pharmacy). Both are important factors when benchmarking a triage recommendation by an OSC to a gold standard.

A major challenge with producing a consensus in the clinical evaluation process for OSCs or in comparing the performance of different OSCs is that different OSCs use different urgency and setting categories [12]. Whereas some OSCs may have urgency categories with a time horizon of ‘within 1 hour,’ ‘within 1 day,’ and ‘within 1 week,’ others may use ‘within 6 hours,’ within ‘48 hours,’ and ‘within 2 weeks.’ The same issue applies to the speciality or service setting (eg, some OSCs may suggest pharmacist, dentist, and physiotherapist, whereas others may suggest self-care, GP, and ED). This has led to attempts to map the outcomes from different OSCs to variable reference category sets in comparative studies [5,6,11,12]. Guidance is required to standardize the method of comparing outcomes from different OSCs or to specify the use of a standardized triage category set for both service settings and time horizons. Health systems vary considerably in terms of access to health assessment and advice; therefore, triage recommendations that are appropriate in one country or setting may be unrealistic or unachievable for users in other countries or settings. This will need to be considered in the formation of an evaluation standard.

The use of triage ranges should also be considered when benchmarking OSC performance. Several comparative studies assessed OSCs based on whether they exactly matched a gold standard triage category [1,5,6,12]; however, OSC triage outcomes that are slightly outside of the gold standard may still be clinically appropriate and safe [11]. An example case is as follows: a case vignette describes a patient with ear pain. The gold standard triage solution has been set to ‘see GP within 3 weeks.’ When tested, the OSC triage recommendation was ‘see a pharmacist within 1 week.’

In the example described, this OSC triage recommendation does not exactly match the correct gold standard triage solution; however, it may still be considered safe and would likely result in the appropriate use of health resources. It may therefore be more appropriate for a clinical evaluation standard to outline an approach that encourages the use of a gold standard range for triage solutions of both urgency and setting rather than a singular outcome.

## Differential Diagnosis

Benchmarking OSC differential diagnoses to a set of gold standard diagnoses presents unique challenges. The method employed in most published evaluation studies involves using vignettes that are written to represent specific diseases. OSCs are then assessed to see if they suggest this disease as the most likely diagnosis or as part of a differential diagnosis list [1,4,5,11,12].

One significant limitation with this approach is that many OSCs suggest several possible diagnoses, and it is important that each proposed diagnosis is congruent with the case vignette. Secondly, this method is limited when delineating rare conditions from a vignette. This is largely because the symptoms of rare conditions are often also shared with much more common conditions, implying that the ‘ideal’ outcome for a vignette for a rare disease would not necessarily place the rare disease as the top differential. Ideally, all the OSC differentials should be included in a comparison to gold standard differential diagnoses solutions rather than simply matching a specific disease label.

## Safety and Accuracy Thresholds

OSCs are unlikely to always match a gold standard solution exactly. Accepted safety and accuracy thresholds when compared to a gold standard solution, and how such standards should be calculated, will need to be carefully considered in the development of a shared clinical evaluation standard.

In the absence of an agreed standard, developers can set their own safety and accuracy thresholds, which could risk unsafe products being released and causing patient harm. On the other hand, due to concerns about patient safety and product liability, there is also a tendency for OSCs to be risk averse. Studies have demonstrated that OSCs often advise contact with health care services for conditions that can be self-managed and thus ‘overtriage’ patients, which could result in an increased burden on health care systems [1,2,4,13,21-23]. Overtriage may also cause unintended harm to patients through heightened anxiety as well as unnecessary investigations and treatments. As such, it is important that the frequency of OSC outcomes that exceed an agreed gold standard triage or diagnosis severity is considered alongside the frequency of outcomes that fall below it. This is supported by the Care Quality Commission regulatory sandbox on digital health triage tools, which suggested that

assessments should be based on where people have been wrongly escalated resulting in undue anxiety, as well as where tools have failed to address people’s ill health. [10]

## Safety Netting

Some OSCs offer safety netting advice to users in addition to triage and differential diagnosis outcomes. Safety netting includes advice about possible future symptoms that may suggest deterioration or warrant a more urgent health care review. For example, an OSC might suggest that a patient books a routine appointment with their GP within 3 weeks, while also advising that if certain symptoms develop or worsen, they should see a GP sooner or attend ED.

The presence and quality of safety netting is often overlooked in OSC comparative studies, but it is an essential part of traditional doctor-patient consultations and considered during assessments of medical negligence [24]. A consensus clinical evaluation guideline should specify how safety netting should be incorporated into safety and efficacy ratings.

## Inputters

Comparative studies showed that inputters can get different consultation outcomes when testing the same vignettes demonstrated by high levels of interrater variability [12,25].

This may be due to variations in how inputters answer OSC questions. For example, one person’s interpretation of ‘fever’ or ‘severe pain’ may vary from another, causing them to answer questions differently. Research has also shown that inputters may also enter symptoms in a different order, and some may enter an incomplete list of symptoms, both of which can result in completely different OSC outcomes [26].

Comparative studies published to date have used variable numbers of inputters; some have used a single inputter [4-6], while others have used multiple [11,12]. The inputters have also varied in terms of medical literacy, with some studies using qualified medical professionals as inputters [11] and others using nonmedically qualified individuals [4-6,12]. These differences may cause significant variations in vignette interpretation and OSC outcomes. Multiple nonmedically qualified inputters may best represent real-world OSC users.

Given this variation in outcomes depending on individual inputters, a clinical evaluation standard should specify the minimum scores for interrater reliability. This could be combined with stipulating how many independent inputters should be required to test each vignette during evaluation with an average taken of the various obtained outcomes [12]. A defined order for symptom entry and a process evaluating the wording of OSC questions for clarity and ease of interpretation could also be considered.

## Recommendations for Future Work

### Third Party Benchmarking

In addition to shared clinical evaluation guidelines, an important next step in improving confidence in the safety and accuracy of OSCs would be the development of an objective third-party benchmarking process for OSCs. This has been recommended by the Care Quality Commission sandbox, stating the following:

NHSX and NHS England should work with NICE NHS Digital to develop and publish the results of a fair test of clinical performance. [10]

The results would ideally involve the curation of a set of evaluation vignettes described as a “national dataset of real patient histories, which is not shared with suppliers” [10].

Independent case vignette repositories have also been suggested by authors of comparative studies [6].

Two United Nations agencies—the World Health Organization and the International Telecommunication Union—established a Focus Group on Artificial Intelligence for Health (FG-AI4H) in July 2018. FG-AI4H is developing a benchmarking process for health artificial intelligence models that can act as an international, independent, standard evaluation framework. It has a topic group focused on artificial intelligence–based symptom checkers with participation from numerous OSC developers, including Ada, Healthily, Babylon, and Buoy Health [18]. As with a clinical evaluation standard, it will be essential that this process can keep pace with rapid development of digital products and does not become a barrier to innovation.

### Protocols for Prospective Real-world Evidence

The clinical evaluation methods described in this paper relate to a theoretical validation of a model’s performance that would often be performed by developers prior to product release or during the release of product updates. This should be distinguished from prospective clinical trials of OSCs in real-world settings. There is a strong need for studies of the real-world impact of OSCs on health care systems [7,14,23]. Robust prospective clinical studies comparing OSCs to existing provision, conducted by independent researchers, will be required in the ‘preprimary care’ and community setting to obtain a complete assessment of clinical product performance [27].

Some prospective clinical studies have been conducted to date comparing OSC triage to laypersons [28,29] and comparing OSC diagnoses to clinician diagnosis in real-world patients [30-32]. However, as with vignette-based evaluation studies, these prospective clinical studies demonstrate significant methodological variation, including the methods used for determining a gold standard outcome and benchmarking to this standard. Therefore, in addition to a standardized OSC vignette-based clinical evaluation process, published protocols with standardized methods specific to prospective clinical studies would also be helpful. Conducting prospective trials at a pace that matches rapid iteration of products will present novel challenges and will require innovative approaches to evaluation methodology.

Guidance is also required on how to evaluate the extent to which OSCs’ advice can be trusted and how user behavior varies compared to when they are given advice from health care professionals, such as triage nurses, pharmacists, or GPs [23]. Compliance with OSC advice is expected to be relatively low; evaluation of the NHS Pathways algorithm suggested that 30% of users who are told to attend emergency department using the algorithm do not comply. Conversely, 10.8% of users attend emergency department when they are advised against it [21].

User satisfaction as well as product usability and acceptability should also be further investigated. Some studies of usability of individual OSCs in real-world settings have been published [21,33], but further studies are required. This should include significant patient and public engagement and the exploration of differences among user sociodemographic groups that could impact health care inequality.

## Conclusions

OSCs have significant potential to support the ability of individuals to self-care providing access to quality-assured health care information, and triaging recommendations. The use of these tools at scale could improve the rational use of scarce health resources, while also prompting patients with ‘red flag’ symptoms to seek emergency care promptly. However, there is currently no standardized way of clinically evaluating OSCs or benchmarking accuracy and safety. This makes comparison of OSC performance challenging and raises concerns about risks to patient safety and increasing health care system demand due to the use of OSCs. A set of objective guidelines for vignette-based clinical evaluation is required to instill confidence that an OSC is providing accurate and safe advice without adversely impacting health care systems.

The recommended requirements for a vignette-based OSC clinical evaluation standard summarized in Table 1 can help OSC developers, regulators, and health care systems work together to develop an effective validation standard. A clinical evaluation standard must be able to keep pace with the rapid iteration and development cycles of such technologies. Therefore, it will be essential that it is practical, pragmatic, and dynamic and does not introduce unnecessary barriers to innovation. The manual entry of vignettes that is often used in comparative studies is unlikely to be scalable, and therefore, the standard should also incorporate automated clinical evaluation methods.

The relative roles of vignette-based clinical evaluation versus prospective clinical studies will require further consideration. The rapid iteration of OSCs will likely make it unrealistic (due to both time and financial constraints) for prospective clinical studies to be conducted each time an OSC model is updated. Therefore, vignette-based evaluation is likely to continue to have a significant ongoing role in the validation of OSCs.

In future, the clinical evaluation of OSCs is expected to involve a mixture of vignette-based clinical evaluation by developers, third-party benchmarking, and prospective clinical studies. Therefore, alongside efforts to develop a clinical evaluation standard, the development of a third-party benchmarking process and the publication of protocols for prospective clinical studies to evaluate OSCs in real-world settings are of high priority.

## Abbreviations

FG-AI4H: Focus Group on Artificial Intelligence for Health
GP: General Practitioner
NHS: National Health Service
NICE: National institute for Health and Care Excellence
OSC: online symptom checker
